# Review: The Key Factors to Melanomagenesis

**DOI:** 10.3390/life13010181

**Published:** 2023-01-08

**Authors:** Cristina-Raluca (Jitian) Mihulecea, Maria Rotaru

**Affiliations:** 1 Doctoral Studies, “Victor Babeș” University of Medicine and Pharmacy of Timișoara, 300041 Timișoara, Romania; 2 Dermatology Clinic, Emergency Clinical County Hospital of Sibiu, 550245 Sibiu, Romania; 3 Dermatology Department, Faculty of Medicine, “Lucian Blaga” University of Sibiu, 550169 Sibiu, Romania

**Keywords:** nevi, melanoma, genetic tests, molecular tests, review

## Abstract

Melanoma is the most dangerous form of skin cancer that develops from the malignant transformation of the melanocytes located in the basal layer of the epidermis (cutaneous melanoma). Melanocytes may also be found in the meninges, eyes, ears, gastrointestinal tract, genito-urinary system, or other mucosal surfaces (mucosal melanoma). Melanoma is caused by an uncontrolled proliferation of melanocytes, that at first may form a benign lesion (nevogenesis), but in time, it may transition to melanoma, determining what it is named, melanomagenesis. Some tumors may appear spontaneously (de novo melanoma) or on preexisting lesions (nevus-associated melanoma). The exact cause of melanoma may not be fully understood yet, but there are some factors that initiate and promote this malignant process. This study aims to provide a summary of the latest articles regarding the key factors that may lead to melanomagenesis. The secondary objectives are to reveal the relationship between nevi and melanoma, to understand the cause of “de novo” and “nevus-associated melanoma” and highlight the differences between these subtypes.

## 1. Introduction

Worldwide, melanoma is considered as one of the most aggressive forms of skin cancer, that affects individuals of any age. Melanoma develops from the malignant transformation of the melanocytes located in the basal layer of the epidermis (cutaneous melanoma) [1]. Melanocytes may also be found in the meninges, eyes, ears, gastrointestinal tract, genito-urinary system, or other mucosal surfaces (mucosal melanoma) [1]. Certain studies attest to a linear progression, from nevi, atypical nevi, to melanoma, that may occur under certain mutational factors, although the majority of melanomas may develop de novo (spontaneously) [2]. One of the most important factors known to activate melanomagenesis is exposure to ultraviolet light [3,4], which affects the tumoral DNA causing genetic mutations that could lead to nevi’s progression to melanoma, through an abnormal proliferation of melanocytes [5]. Melanoma is known for its genomic instability and is one of the cancers with the most somatic mutations [4].

This study aims to provide a summary of the latest articles regarding the factors that lead to melanomagenesis, to get a better understanding of this cancer’s biology. The secondary objectives are to reveal the relationship between nevi and melanoma, to understand the cause of “de novo” and “nevus-associated melanoma” and highlight the differences between these subtypes.

## 2. Materials and Methods

We analyzed over 90 of the most recent studies regarding the key factors responsible for the occurrence of melanoma, to provide a review of the possible causes of this neoplasia. We also assessed the relationship between nevi and melanoma by studying the latest articles on this topic, to understand the cause of “de novo” and “nevus-associated melanoma” and highlight the differences between these subtypes.

## 3. Results

Melanoma originates from melanocytes, neural crest-derived cells, that can be found in the skin, eye, and other tissues (ex. meninges, anogenital tract) [6]. The main function of melanocytes is to produce melanin and provide it to the keratinocytes, to absorb UV radiation and protect the keratinocyte’s nucleus from the DNA damage induced by ultraviolet radiation. The data collected from the studied articles were separated into three categories, as follows: Section 3.1 the key factors to melanomagenesis; Section 3.2 from nevi to melanoma—a linear progression; Section 3.3 de novo vs. nevus-associated melanoma.

### 3.1. The Key Factors to Melanomagenesis

Melanoma can be classified based on: 1. the relationship between sun exposure and the location of the primary tumor (melanoma on skin with/without chronic sun damage (CSD)—CSD or non-CSD melanoma); acral melanoma—located on palm, soles, or nail bed; and mucosal melanoma); and 2. the tumoral growth pattern: superficial spreading melanoma (SSM), nodular melanoma (NM), lentigo maligna/melanoma (LM/LMM), and acral lentiginous melanoma (ALM) [7].

The exact cause of melanoma is not yet understood, but there are certain factors that may initiate and promote its development: ultraviolet (UV) radiation, indoor tanning [8,9,10], prolonged sun exposure/sunburn [11], burn scars [12,13,14], pesticides, genetic factors/heredity, geographical location, skin phototype, immunosuppression, hormonal changes, a high number of nevi, neuroendocrine factors, stress, depression [15], trauma, low socioeconomic status, non-melanoma skin cancers, autoimmune diseases, viral infections, biological/cytologic factors, smoking/alcohol, medicines, gender (melanoma has a higher incidence in men [16,17,18,19,20]), etc. [1,21,22,23]. The impact of a part of these factors will be explained in the following lines, see Table 1—the key factors to melanomagenesis.

#### 3.1.1. UV Radiation

Exposure to UV radiation (UVR) is considered the main risk factor for melanomagenesis [1]. Both natural and artificial lighting systems are sources of UV radiation [1]. Melanocytes are known for their photoprotective function (they protect the nuclear DNA and reduce its damage) as a response to UV exposure through melanin synthesis [24]. The melanin pigment has a crucial role in the protection against the effects of UV radiation and other environmental factors [25]. While melanin protects against skin neoplasms, its presence is necessary for the oncogenic transformation of melanocytes, as melanogenesis may show mutagenic activities, which can lead to the initiation and progression of melanoma. The synthesis of this pigment is modulated by sun exposure and hormonal factors [25]. Melanin is then transferred to keratinocytes that cause tanning, due to UV exposure [24]. Some studies attest that melanoma is inversely related to skin pigmentation, having a lower incidence in individuals with dark skin tones and a higher incidence in individuals with fair skin tones [24].

Approximately 90% of melanomas are caused by ultraviolet radiation from sunlight, which induces DNA damage, leading to DNA mutations and contributing to melanomagenesis [24]. UV radiation can be classified into three categories: ultraviolet C (UVC; 200–290 nm), ultraviolet B (UVB; 290–320 nm), and ultraviolet A (UVA; 320–400 nm) [24]. UVB is the main cause of sunburns, it damages the epidermis, and has a central role in skin neoplasms. UVB waves are absorbed by the nuclear proteins and acids of the cutaneous cells [1]. The irreparable DNA damage promotes the onset of somatic mutations, with the transformation of normal cells into oncogenic cells [1]. UVA penetrates the skin deeper than UVB/UVC but is mainly responsible for its photoaging effect [24]. UV damages the skin cells and tissues and forms DNA mutations, altering the DNA integrity, which impairs many tumor-suppressing genes [24]. UV radiation may also suppress immunity: it inhibits antigen presentation, stimulates the release of immunosuppressive cytokines (ex. TNF-α), and determines the apoptosis of immune cells [24]. The immunity of the skin depends on the functioning of epidermal Langerhans cells (LCs)—the main antigen-presenting cells (APCs) of the skin. UV also damages LCs, decreasing their numbers and inhibiting their antigen-presenting function [24].

All this considered, exposure to UV radiation either from sunlight or tanning beds contributes to cellular DNA damage, oxidative stress, immunosuppression, and skin inflammation, having a major role in melanomagenesis [26].

#### 3.1.2. Genetic Factors

Melanoma is a neoplasm with complex pathogenesis, due to the genetic mutations within the molecular pathways that control cell proliferation, differentiation, and survival [7]. UV radiation is the most prevalent factor in determining somatic mutations that may cause the occurrence of melanoma [3]. Somatic mutations are produced by multiple mutational processes, which generate specific mutational signatures [4,26]. The most frequent mutational signatures found in melanoma were: signature 1B (associated with age and the spontaneous deamination of 5-methyl-cytosine), signature 7 (UV radiation-induced mutations), and signature 11 (associated with patients treated with the alkylating agent temozolomide) [3].

As for the genetic mutations, *BRAF^V600E^* is one of the most common mutations in melanoma (60% harbor this mutation) development [5]. This mutation activates the mitogen-activated protein kinase (*MAPK*) pathway and causes bursts of melanocytic proliferation followed by growth inhibition and senescence, an event known as oncogene-induced senescence, with senescence escape being thought of as a cause of melanomagenesis [5]. Epigenetic alterations and the loss of tumor suppressor *PTEN* may also contribute to senescence escape [5]. The majority of BRAF mutations are represented by the substitution of valine at position 600 (V600) [7]. 75% of the BRAF variants are represented by the V600E mutation, 19% by the V600K mutation, and only 6% by V600D/V600R mutations [7].

Other mutations that contribute to melanomagenesis are: RAS (ex. TACC1, CTNNB1), and non-BRAF/non-RAS (ex. KIT, CDK4, MITF, CCND1, TERT, CDKN2A, WT1, EZH2, STK19, PIK3CA, RASA2, SNX31, FBXW7, PREX2, SF3B1, RB1, IDH1, MAPK2K1-2, DDX3X, RAC1, PPP6C, ARID2, NF1, TP53) [26,27,28,29,30,31,32]. TP53 and PTEN mutations are mostly found in advanced/invasive melanomas [33]. NRAS, HRAS, BRAF, and GNAQ, have also been identified in benign nevi, and their presence is associated with congenital nevi, Spitz, acquired, and blue nevi [34,35].

The characterization of melanoma molecular subtypes is very important for the right therapeutic decision. Melanoma can be classified into the following molecular subtypes [1,26,36]:

1. MAPK subtype: in melanoma patients, the MAPK (mitogen-activated protein kinase) pathway, is hyperactivated causing a cascade activation of NRAS, BRAF, MEK, and ERK (MAPK), intensifying the transcription of the genes involved in cell proliferation, growth, and mutation [1]. The BRAF kinase has a role in the regulation of the signaling pathway between MAPK and ERK (extracellular signal-regulated kinase), which controls cell division and differentiation [1]. The BRAF gene mutations cause uncontrolled cell division of the melanocytes and the development of melanoma [1]. BRAF mutations are often found in common or atypical nevi but are not enough to cause the occurrence of melanoma [1]. The most common mutation in BRAF is the conversion of thymidine to adenine (T→A), resulting in the substitution of valine with glutamate (V600E) [1]. The MAPK subtype with BRAF mutations (found in approximately 50% of melanomas, mostly in younger patients and in non-CSD melanoma) can be classified as follows:

(a) With the activation of the PI3K–AKT–mTOR pathway, increased AKT3 expression and/or the loss of PTEN. PI3K-AKT is involved in the modulation of cell survival, growth, and apoptosis. The increased activation of PI3K signaling is seen in melanomas triggered by the mutations, deletions, and promoter methylation of the coding genes of the PTEN inhibitor [1]. PTEN encodes a tumor suppressor protein that cannot fulfill its role if it is damaged, causing continuous cell proliferation (this gene is also involved in cell division by keeping cells from growing and dividing) [1,36]. mTOR is an activated protein found in 73% of human melanoma cell lines [1]. Therefore, this subtype can benefit from treatment with PI3K, AKT, and mTOR inhibitors [26]. A study by Leonardi et al. shows that the MAPK-pathway (mitogen-activated protein kinase) and PI3K-pathway (phosphoinositol-3-kinase) promote melanomagenesis through various genomic mutations on the components of these pathways [37].

(b) Alteration of the p16^CDKN2A^–CDK4–RB pathway, with the inhibition of p16^CDKN2A^ and/or amplification of CDK4. CDKN2A (cyclin-dependent kinase inhibitor 2A) encodes two proteins—p16^CDKN2A^ and p14^CDKN2A^. In normal conditions, p16^CDKN2A^ inhibits CDK4 (protein kinase cyclin-dependent kinase 4)/ CCND1 (cyclin D1) affecting the cell-cycle progression which depends on the retinoblastoma susceptibility protein (RB) [26]. In melanoma, p16^CDKN2A^ is inactivated and CCND1 amplificated due to various epi/genetic mechanisms [26]. P16 acts as a CDK inhibitor, slowing the progression of the cell cycle [36]. On the other hand, p14^CDKN2A^ normally prevents the degradation of P53 and favors its control of cell-cycle progression [26]. In melanoma, P53 can be inactivated due to the mutations of p14^CDKN2A^ [26]. Taking all this into consideration, these patients may be treated with CDK4/6 inhibitors [26]. CDK4 is known for contributing to the regulation of the cell cycle, triggering metastasis, and interfering in the phosphorylation of RB [36].

(c) MITF gene amplification. MITF plays the most relevant role in melanoma, by controlling the differentiation and proliferation of the melanocytes [26]. A low/ absent expression of MITF leads to apoptosis, while overexpression determines cell differentiation and an anti-proliferative effect to some extent [26]. Patients could benefit from treatment with histone deacetylase (HDAC) inhibitors as they may interfere with the expression of MITF protein [26].

2. NRAS subtype: NRAS mutations (found in 15–20% of melanomas, but rare in nevi [1]; they have a more aggressive clinical course) are caused by prolonged sun exposure, mostly found in nodular melanomas and melanomas with a Breslow index of over 1 mm [1]. This mutation is important in the initiation and promotion of neoplasms, associated with possible activation of the PI3K-AKT pathway, therefore PI3K or MEK inhibitors treatment could be suitable in this case [26]. NRAS mutations result in a serial activation of serine/threonine kinases, stimulating cell cycle progression, transformation, and survival. This may be caused by the hyperactivation of either growth factor receptors like c-Met (tyrosine-protein kinase Met), EGFR (epidermal growth factor), c-KIT (tyrosine kinase receptor), or by the functional loss of the NF1 (neurofibromatosis type 1) gene [1].

3. cKIT subtype: cKIT (tyrosine kinase receptor) has an important role in cell differentiation/proliferation, melanocyte development, tumor formation, development, migration, and recurrence [26,36]. Its mutations could cause insufficient pigmentation and can activate multiple signaling pathways, especially the PI3K-AKT and MAPK pathways [1,36]. It is more frequently found in acral (10%), mucosal (15–20%), and CSD (5%) melanomas [1]. All this considered, the cKIT inhibitors could be utilized in the presence of certain activating mutations [26].

4. GNAQ/GNA11 subtype: GNAQ/GNA11 mutations are found in 80–90% of uveal melanomas [38]. These genes encode the alpha subunits of the heterotrimeric G proteins (Gq/G11) and their mutations activate various signaling pathways, acting as driver genes in the oncogenic process [38]. Patients may be treated with MEK inhibitors since these genes’ activation leads to the activation of the MAPK pathway with MEK enzymes being one of its main effectors [26].

A family history of melanoma is associated with an increased risk due to shared genetic mutations and/or lifestyle habits (ex. outdoor hobbies, sun exposure, etc.) [39]. There are certain inherited genes (*CDKN2A, CDK4, MITF, TP53, XPC, XPD, XPA, TERT, POT1, ACD, TERF2IP, BRCA 1/2, BAP 1,* and *PTEN*) whose mutations may be causing hereditary melanoma [1]. For example, *CDKN2A* mutations (located on chromosome 9) are found in 3-20% of the families with a history of melanoma [1,39]. When this gene suffers mutations there is a loss of cancer suppression and it leads to uncontrolled cancer cell proliferation [39]. Patients with familial atypical multiple mole melanoma (FAMMM) syndrome have a high risk for melanoma development [40]. Patients with FAMMM syndrome have a 10.7% risk of melanoma and a higher risk of melanoma also depends on the number of family members affected (approximately 100% risk if two or more relatives have atypical nevi and melanoma) [21].

**Table 1:** The key factors to melanomagenesis, [1,3,4,5,7,26,34,35,37,39], UV = ultraviolet, UVA = ultraviolet A, UVB = ultraviolet B, HIV = human immunodeficiency virus, AIDS = acquired immunodeficiency syndrome.

Melanoma: Trigger Factors				
UV Radiation	Genetic Mutations	Skin Phototypes	Immunosuppresion	Other Factors
Sunlight (UVA, UVB) Indoor tanning	BRAF^V600E^ Other mutations: PTEN, RAS, TP53, NRAS, HRAS, GNAQ, CDKN2A, CDK4	I/II—fair skin, blonde/red hair, a high number of freckles, blue/green eyes.	HIV/AIDS Immunosuppressive therapies	Inflammation Autoimmune diseases Metabolic syndrome Hormonal factors Aging Stress

Aside from genetic mutations, epigenetics is another important factor involved in melanomagenesis [36]. Epigenetics focuses on the change of the gene’s functions that are meiotically and/or mitotically inherited [36]. Epigenetic factors modify the expression of microRNAs, and they target genes involved in cell differentiation, and growth/death, having a role in melanoma progression [36]. The main mechanisms of epigenetics identified in melanoma are: chromatin modifications (DNA methylation), histone alterations (they cause post-transcriptional modifications altering the chromatin state, useful for cancer progression), modifications of histones acetyl and methyl groups (they affect cancer progress, modulate the response to anticancer drugs and have a critical role in melanoma’s signaling pathways), and the noncoding RNAs/microRNAs (involved in melanoma genesis, cell cycle regulation, tumoral growth, cell invasion/migration/apoptosis and drug resistance) [36]. All this considered, epigenetics mechanisms should be furtherly studied, as they could predict the outcome of certain melanoma therapies [36].

#### 3.1.3. Skin Phototypes

There are six types of skin phototypes, with I as the lightest and VI being the darkest. Individuals with skin phototypes I/II have fair skin, blonde/red hair, a high number of freckles, and blue/green eyes, making these two phototypes the most sensitive to UV exposure [1]. These patients are more prone to develop melanoma, as they have a lower resistance to UVB rays [1].

#### 3.1.4. Immunosuppression

Patients with immunosuppressive conditions, such as AIDS/HIV are predisposed to melanomagenesis as prolonged immunosuppression cannot protect the individual from the onset or progression of melanoma or other neoplasms [1]. The systemic immunosuppression caused by agents like cyclosporine in renal transplantation patients determines the inhibition of tumor suppressor factors like p53 and the activation of proto-oncogenes (HRAS, KRAS, or NRAS) [1]. This causes DNA damage and somatic mutations, inducing the onset and progression of melanoma [1]. If cutaneous melanoma occurs in patients with an intact immune system, it will develop mechanisms to evade the body’s immune response, which includes the activation of immunosuppressive cytokines that will further modulate antigen-specific immune cells to initiate tumorigenesis [41,42].

#### 3.1.5. Autoimmune Diseases/Inflammation

Chronic inflammation is recognized for its capacity to determine epidermal cell transformation and malignant progression, contributing to approximately 20% of all human cancers, including melanoma [43]. Under various factors (for example, trauma, and infections) inflammation determines the transformation of cancer-originating cells by producing reactive nitrogen intermediates (RNI) and reactive oxygen species (ROS) that cause DNA damage and genomic instability [43]. Some studies indicate that periostin (a contributing factor in tumorigenesis) and M2 macrophages may play a crucial role in melanoma progression through inflammation [44]. Many pro-inflammatory exogenic factors may promote neoplasm development through the nuclear factor-κB (NF-κB) and STAT3 signaling pathways [45]. Increased levels of miR-21(an inhibitor of key tumor suppressors) are associated with inflammation, for example, in diabetes mellitus (type 1 and 2), atopic conditions, and chronic renal fibrosis [45]. Chronic inflammation may thus promote melanomagenesis, through a high level of miR-21. There are reports that neutrophilic cutaneous inflammation promotes angiotropism and metastatic spread of melanoma [46].

As for autoimmune diseases, some studies show that in type 1 diabetes mellitus, there is an increased response to inflammatory cytokines, demonstrating that this type of diabetes T1DM might be involved in melanoma development, through chronic inflammation [45]. In the literature, it is noted that psoriasis patients have a higher risk of developing certain cancers, due to the inflammation that these patients have [47]. Regarding psoriasis’ therapy it seems that the biological treatments do not necessarily raise the risk for melanoma development and are considered safe [48,49]. One of the most common chronic inflammatory skin diseases is atopic dermatitis (AD) and there is a lot of controversy regarding the extent of skin cancer risk that these patients have [50]. These patients have a hyperactive immune system, which could prevent skin cancers [50]. Some studies, also report that patients with AD have fewer nevi, which implies that this pathology could prevent melanoma, as a higher count of nevi is considered a risk factor for developing it [50]. Marasigan et al. report that there is an inverse correlation between atopy and melanoma and that there is not enough evidence that supports an association between AD and melanoma progression or survival [50]. While some studies attest that there is a negative or inverse association between atopy and melanoma, other studies report that AD patients could have a high risk of developing it [50]. Since there is no strong scientific evidence, AD patients should use photoprotection, avoid excessive exposure to the sun, and monitor their nevi by self-examination and regular dermatologist appointments.

Considering the role of inflammation in tumor initiation, promotion/progression, angiogenesis, and metastasis, inflammatory pathways may become future targets for melanoma prevention [51,52,53].

#### 3.1.6. Other Factors

Aside from the factors listed above, there are also other factors associated with melanoma development, such as the microenvironment, metabolic syndromes, hormonal factors, aging, etc.

The onset of melanoma could be associated, through direct/indirect interactions, with the *cancer microenvironment* formed by different types of cells (fibroblasts, macrophages, lymphocytes, keratinocytes, other immune system cells, adipocytes, and cells that form the cutaneous blood vessels) that interact with each other through signaling proteins and cytokines [54]. Studies examining the microenvironment of melanomas and macrophages associated with tumors have shown that they may be involved in all stages of melanomagenesis: in neoplasm initiation, they determine an inflammatory microenvironment and suppress the antitumoral activity of the immune system, they stimulate angiogenesis, enhance migration/invasion of the oncogenic cells, and are involved in the metastatic process [54]. Some studies suggest that keratinocytes and fibroblasts may contribute to melanomagenesis, as they inhibit certain mechanisms that prevent uncontrolled melanocytic proliferation [5]. Keratinocytes inhibit senescence-related genes, promote the uncontrolled proliferation of melanocytes, and affect the balance of the melanocyte-secreted factors that aid in the process of tumorigenesis [5].

Melanin biosynthesis and reactive oxygen species (ROS)—Melanogenesis is a process during which melanin is synthesized and distributed [55]. There are several factors that are known for modulating melanogenesis, such as UVR, MITF (microphthalmia-associated transcription factor), the ERK/MAPK pathway, immune regulation, or mitochondrial dynamics [55]. The main function of melanin is photoprotection by absorbing UVR, but it can also lead to high levels of intracellular ROS (reactive oxygen species) increasing melanoma susceptibility [55]. UVR is responsible for increasing ROS levels in the melanocytes and keratinocytes causing DNA damage and impairing the natural antioxidant defenses, eventually leading to melanomagenesis [55]. Melanin is known for its protective effect against reactive oxygen species and toxic radicals [56]. However, melanin biosynthesis has the potential to produce reactive oxygen species that determine DNA damage and the malignant transformation of the melanocytes [56]. Oxidative stress (OS) has been reported to be involved in all the phases of melanoma (from genesis to metastasis), it may alter gene expression by inhibiting key epigenetic enzymes’ interaction with the DNA and it also determines chemoresistance [55,57]. This may lead to genomic instability and global hypomethylation [55]. ROS-induced abnormal DNA methylation pattern alterations can determine malignant transformation and cancer progression [55]. ROS may be generated by the increased metabolism of transformed cells, enzyme activity, UVR, melanin production, an immune reaction against tumors, an altered oxidant system, and exogenous factors (UVR, air pollution, ozone, infections, chemicals, cosmetics, drugs, toxins, or non-ionizing radiation) [55,57]. Additionally, the production of hydrogen peroxide and the consumption of reduced glutathione (GSH) during melanin biogenesis are responsible for a high level of ROS in melanoma [55]. Despite the skin’s antioxidant defense mechanisms, excessive ROS production cannot be fully neutralized [57]. A controlled level of ROS is beneficial for the skin’s homeostasis and the epidermal keratinocyte’s proliferation, but excessive levels of ROS may lead to DNA damage, aberrant gene expression, cell death/mutations, injury of the local tissues, cancer progression, and metastasis [55,57].

Persister melanoma cells are considered dormant cells in a mass of cancer cells that can tolerate a high level of drugs [58]. A study by Karki et al. attests that persister cells could be induced by chemotherapy [58]. These cells experience metabolic alteration to improve the survival of tumoral cells, sustain cell proliferation, avoid the action of drugs, and fulfill energy requirements [58]. Travnickova et al. report that melanoma persister cells originate from the primary tumor and that they contribute to cancer recurrence and drug resistance [59]. Melanoma is thought to be a chemotherapy-resistant cancer, and this resistance could be attributed to persister cells [58,59]. The further study of persister cells could help develop novel cancer therapeutic strategies.

Metabolic syndrome and melanoma—A patient with metabolic syndrome (formed of at least three of the following: hypertension, impaired glucose tolerance, abdominal obesity, high triglycerides levels, and low HDL levels) may have a higher risk of developing melanoma [45]. Adipocytes activate AKT and mTORC1 in melanoma cells and stimulate their proliferation, migration, and invasion (they promote melanoma aggressiveness through high fatty acids oxidation) [45].

Hormonal factors—There is epidemiological evidence that associates acne, prostate cancer, and melanoma with the androgenic hormones as a possible link [45]. Apparently, patients with positive androgen receptors have a worse survival as opposed to melanoma patients with negative androgen receptors, as they also promote melanoma metastases through MITF signaling [45]. Metastatic melanoma cell lines have also been shown to have an increased level of growth hormone receptor (GHR) [45]. Melanoma metastases occur rapidly and are caused by a complex of factors, out of which genetic mutations are some of the most important factors [60]. The growth hormone influences different oncogenic signaling pathways, especially JAK2-STAT3 [45]. Vitamin D deficiency has also been associated with a poor prognosis in melanoma. A high level of vitamin D is associated with a low tumoral thickness and protects against relapses and death caused by melanoma [45,61]. Some studies suggest that neuroendocrine factors may play a role in melanoma development, influencing the tumoral cells’ proliferation and metastasis capability [62]. For example, catecholamines stimulate proliferation, motility, and invasion of melanoma cells, while glutamate may activate angiogenesis [62].

Aging—it seems that the incidence of melanoma may increase with age and that melanoma in young individuals may be attributed to a complex of genetic factors, multiple nevi, as opposed to the older individuals where exogenic factors play the most important role [63,64,65]. A higher age often leads to a poor prognosis in stages I, II, or III, and is associated with high levels of miR-21, reaching the highest level at around 66 years of age (coinciding with the median age of melanoma incidence) [45].

Smoking/alcohol—Some studies have identified smoking as a predictor of poor outcomes in melanoma patients [45]. It seems that nicotine induces the expression of miR-21 and promotes melanoma cell proliferation and migration [45]. The same goes for pollution, a factor that increases miR-21 expression and activates the PI3K/AKT pathway, leading to melanomagenesis [45]. Cigarette smoking is known to determine premature skin aging, aggravate psoriasis, inhibit wound healing, and may also cause squamous cell carcinoma [22]. Alcohol may also be associated with an increased risk of invasive melanoma with white wine consumption having the highest risk of developing melanoma [66]. It seems that regarding this factor mostly UV-spared sites (trunk) were affected by melanoma [66].

Stress—chronic stress can be an important factor in cancer progression and melanoma spreading [67]. Moreover, catecholamines (stress hormones) may promote the aggression of melanoma cells through the interaction with specific receptors [67]. Some studies state that oxidative stress is a major force involved in all the phases of melanomagenesis, from initiation to the progression of melanoma, up until the onset of the metastases and chemoresistance, making it a target for therapy [55]. All this considered, there is a clear involvement of chronic stress in melanoma initiation and progression [68].

Therapies/medicines—immunomodulating medicines may increase cutaneous photosensitivity and suppress the immune system’s responses, determining a higher melanoma risk [69]. Certain papers report that immunosuppressive agents like methotrexate (MTX)may increase skin cancer risk in patients with psoriasis [48]. As for melanoma suppression, it appears that resveratrol (a natural polyphenolic phytoalexin) has been demonstrated to inhibit melanoma cell viability, migration, and invasion, blocking melanoma progression [70].

Mucosal (oral) melanoma risk factors—Mucosal melanoma is a very rare and aggressive melanoma subtype that arises from the oncogenic transformation of the mucous membranes’ melanocytes [71]. This type of melanoma is biologically, epidemiologically, and molecularly different from cutaneous melanoma [72]. The risk factors and causes of mucosal melanoma’s development are not fully understood and there is little information regarding its molecular markers [71]. Mucous melanoma develops most frequently in the head and neck cavities (including the oral/nasal cavity and accessory sinuses), followed by the rectum/anus and the feminine genital tract [72]. Oral melanoma often presents clinically as heavily melanin-pigmented lesions but may also appear amelanotic [56]. The prevalence of this type of melanoma increases with age, affecting both females and males aged between 40 and 70 years [73]. It rarely affects the white population and is most often found in black, Japanese, and Indian individuals [73]. Unlike cutaneous melanoma, oral melanoma is not related to UVR exposure [73]. This type of melanoma may appear de novo, but approximately 30-37% of them occur on preexisting lesions possibly under the influence of various factors (denture irritations, cigarette smoking, infections) [56,73]. As of yet, oral melanoma is not associated with any confirmed oncogenic agents [56]. About 30% of oral melanomas develop on hyperpigmented melanin lesions which may lead to the conclusion that dysregulation of melanin biosynthesis, its products (reactive oxidative species (ROS), intermediates—semi/quinones, which are potentially mutagenic and may promote cytogenetic instability) or a loss of the melanosomal membrane’s integrity, with the leakage of toxic melanin particles, could have a role in the carcinogenic transformation of melanocytes [56]. Regarding de novo oral mucosal melanoma, it is not understood if it is caused by an overproduction of melanin as an early/late event in melanomagenesis due to the acquisition of a malignant phenotype [56]. All this considered, melanin seems to be a risk factor for melanoma development [56]. As for the genetic mutations and oral melanoma’s molecular pathways, the following are reported as risk factors for its development [56]: (a) the gain-of-function mutations in KIT protooncogene (dysregulated cKIT intracellular signaling pathways—PI3K, JAK/STAT, Ras-Raf-MAPK pathway which causes increased cell survival and proliferation) [56]; (b) MC1R polymorphism—determines damaged DNA repair mechanisms, promotion of toxic melanin particles synthesis, and increased survival and proliferation of the melanocytes [56]. Normally, MC1R (melanocortin receptor 1) regulates the proliferation and survival of melanocytes and promotes DNA repair in case of damages caused by oxidative stress [56]; and (c) the altered expression of cell adhesion molecules, which causes the downregulation of E-cadherin and the upregulation of N-cadherin that contribute to the increase of migration, proliferation, and invasivity of melanoma cells [56].

### 3.2. From Nevi to Melanoma—A Linear Progression?

There is a genetic overlap between nevi and melanoma, having a shared environmental influence of UV radiation, yet nevi remain localized, while melanoma can spread from its primary location to distant organs [74]. Some studies attest that there may be a linear progression from nevi to melanoma under certain mutational factors [2]. To confirm this theory, a study by Damsky et al. states that 33% of melanomas could be derived from nevi [75], while a study by Sondermann et al. suggests that 30-50% of melanomas and more than half of melanomas in young patients evolve from benign lesions, and neither clinicians nor artificial intelligence (AI) algorithms are yet able to predict a nevus’ oncologic transformation [76]. Additionally, the Clark model of melanoma pathogenesis attests that there are certain steps that occur during a progression from normal melanocytes to melanoma cells [40]. These steps include the formation of common nevi, then atypical nevi, then melanoma in situ, and later, invasive melanoma. This linear progression is thought to be driven by the accumulation of genetic mutations and epigenetic changes [40]. A study by Eddy et al. [77] suggests that melanomagenesis is based on the following phases: 1. normal melanocytes acquire an initiating driver mutation that causes melanocyte hyperplasia and nevi development (breakthrough phase, with a low number of mutations); 2. in the expansion phase some of the nevi progress into atypical nevi and later into melanoma in situ, associated with a high number of mutations; and 3. after the accumulation of numerous mutations (ex. *CDKN2A*, *TP53*, *PTEN*) the primary melanoma goes into an invasive phase [77]. Melanomas that develop on non-sun-exposed anatomical sites are associated mostly with BRAF V600E mutations^,^ which are considered one of the key factors to nevogenesis (formation of common nevi) [6]. The frequency of initiation of the BRAF V600E mutation is influenced by UV radiation, and the patient’s genotype, while the number or morphological appearance of nevi may be influenced by the individual’s phenotype (e.g., fair skin, low tanning ability) [6].

Whether nevi are melanoma precursors remains controversial [78]. Pathology-based studies have found that 20%-30% of melanomas contain nevus cells, suggesting a direct transformation of nevi into melanoma [78]. According to some studies, after acquiring different mutations, the melanocytes will proliferate to form nevi [6,78]. Common nevi develop in the first two decades of life, mainly on sun-exposed anatomical sites (acquired nevi), and may tend to regress after the sixth decade [6]. After the acquisition of certain mutations, another category of melanocytic lesion arises, one that is an intermediate form between common nevi and melanoma–atypical nevi. These lesions have clinical, dermoscopic, and histological similarities with early melanomas [2,24].

Recent studies have shown that common nevi have mainly one driver mutation—BRAF^V600E^, whereas atypicalnevi may have multiple mutations (ex. NRAS and BRAF^nonV600E^) [6]. This contradicts the linear progression theory that common nevi may evolve into atypical nevi, as this type of lesions rarely have BRAF^V600E^ mutations, suggesting that some sporadic atypical nevi may follow a separate evolutionary trajectory [6]. Tschandl et al. report that the presence of BRAF/NRAS mutations does not predict the chance of malignant transformation of a nevus [79]. In this study, the frequency of BRAF mutation was similar in melanoma (63%) and nevi (65.2%) and the BRAF/NRAS transformed nevi did not have a higher chance of being associated with melanoma than a wild type of nevi [79]. This study states that BRAF/NRAS mutations within a nevus do not play a major role in the development of melanoma from nevi [79]. Other studies report that mutational signatures imply that UV radiation-mediated mutagenesis of superficial melanocytes of the nevus is the predominant pathogenic mechanism that drives the progression of nevi to melanoma [6].

A study by Ulanovskaya et al. states the cytosolic enzyme NNMT (nicotinamide N-methyltransferase) promotes migration, invasion, proliferation, and survival of cancer cells and that aggressive cancer lines possess a higher level of NNMT [80]. It is also stated that it supports tumorigenesis and may be used as a potential anticancer target [80]. According to an article by Ganzetti et al., it is reported that NNMT is overexpressed in melanoma, in comparison to nevi, but that there is an inverse relationship between the Breslow index, Clark level, mitoses, and ulceration (prognostic factors) [81]. This idea is also supported by a study made by Campagna et al. [82]. All this considered, the enzyme NNMT could be used as a prognostic biomarker for melanoma, and since it plays a role in tumorigenesis and cancer progression, if encountered in nevi, it could be involved in a linear progression from nevi to melanoma, as under oncogenic factors, nevi may eventually transform into melanoma.

### 3.3. De Novo vs. Nevus-Associated Melanoma

Melanoma may arise “de novo” (spontaneously) or on preexisting lesions (common or atypical nevi). This sustains the theory that under the circumstance of certain mutational factors, there can be a linear progression model, from common nevi, atypical nevi, to melanoma. Melanomas arising from nevi, common or atypical, normally develop intra-epidermally [6].

There are few known facts about the endogenic/exogenic factors that may cause melanoma to arise in normal skin or on a preexisting nevus [78]. Certain studies attest that only 20%–30% of melanomas are “nevus-associated” (NAM), with the majority of melanomas (70–80%) arising spontaneously (de novo—DNM) [78,83,84]. However, some authors report that this might not be the correct percentage of DNM as preexisting nevi may have been overgrown/destroyed by melanoma cells [85]. Studies show that de novo melanomas (DNM) are mostly associated with poorer outcomes and shorter survival compared to nevus-associated melanomas (NAM) [78]. DNM usually have a tumoral thickness greater than 1.0 mm, and are associated with the nodular melanoma subtype located mostly on extremities, a higher stage than stage I, older age onset, a low number of nevi, and tumor ulceration [78]. Some studies highlight that the lentigo maligna melanoma (LMM) subtype is very rarely associated with remnants of nevi [85,86].

It seems that, on histologic examination, nevus-associated melanomas present either as tumors growing on preexisting nevi or as a transformation of a common nevus into an atypical nevus and, later, fully developing into a melanoma [85]. In a study by Pandeya et al., NAM was associated with the superficial spreading melanoma subtype, with a younger age onset, blonde hair, fair skin, green/hazel eye color, no/fewer facial freckles, a high number of nevi, tumors located on the trunk, a tumoral thickness of under 0.5 mm, and BRAF^V600E^ mutation [40,87]. Apparently, patientswith nevus-associated melanomas had many more moles on their skin as teenagers, but with less dermal elastosis as opposed to DNM [87]. These findings may suggest that NAM may arise through a different sequence of causal events, compared to those leading to other types of melanomas [87]. A study by Tas et al. suggests that even though DNM is associated with poor outcomes, the survival rates of DNM and NAM are similar, see Table 2—de novo melanoma vs. nevus-associated melanoma [88].

## 4. Discussion

Melanoma is a skin cancer with a very complex pathogenesis, caused by molecular and genetic mechanisms that are not yet fully understood and it is one of the cancers with the most somatic mutations according to a study by Alexandrov et al. [3,4,26]. Exposure to intense UV radiation, a high number of nevi, heredity, age, and light skin are some of the most important risk factors associated with increased melanoma incidence [43,55,57,89]. It is crucial to avoid all factors that could lead to melanomagenesis, if possible, and especially, to limit sun exposure and use photoprotection products.

According to a study by Conforti et al., although the epidemiological data from 1980–1990 suggest an increase in the incidence of melanoma across all ages, the studies from the last 10 years indicate a 5% reduction in the incidence of melanoma in young individuals (between 15–24 years) [90].

A family history of melanoma is associated with an increased risk due to shared genetic mutations and/or lifestyle habits. A healthy lifestyle may also have a positive influence on reducing the effect of some mutational factors [45]. In a study by Haenssle et al., patients with FAMMM syndrome or/and with multiple melanomas developed less frequently nevus-associated melanoma compared to patients with a high count of common nevi/ no previous melanoma [40]. It is thought that the onset of de novo melanoma in these patients is caused by genetic factors related to the FAMMM syndrome, as genetic analyses often reveal *CDKN2A* mutations associated with this syndrome [40].

Aside from genetic alterations, epigenetics is another factor that could determine the occurrence of melanoma [36]. The main epigenetic mechanisms involved in melanoma are: chromatin modifications, histone alterations (modifications of histones acetyl and methyl groups), and the noncoding RNAs/microRNAs (involved in melanoma genesis, tumoral growth, cell invasion/migration/ apoptosis, and drug resistance) [36]. We consider that epigenetics mechanisms should be furtherly studied, as they could predict the outcome of certain melanoma therapies.

Another important factor involved in melanomagenesis is persister cells. While some studies report that persister cells could be induced by chemotherapy [58], other studies attest that melanoma persister cells originate from the primary tumor and that they contribute to cancer recurrence and drug resistance [59]. These cells go through metabolic alteration to improve the survival of tumoral cells, sustain cell proliferation, avoid the action of drugs, and fulfill energy requirements [58].

The clinicopathological examinations for melanoma diagnosis should be integrated with molecular techniques, to increase the accuracy of melanoma detection and to have a better understanding of its pathogenesis [26]. The introduction of novel techniques for genetic and molecular analyses may lead to a better understanding of melanoma pathogenesis [7]. According to a study by Bastian et al. [91], melanomas may be categorized into multiple biological categories, which differ in types of origin cells, clinical/histologic presentation, the age of onset, the type of metastasis, the ethnic/gender distribution of patients, the role of UV radiation, mutational processes [91].

More research should be conducted to elucidate the clinical relevance of certain melanoma molecular mechanisms, to improve the clinical management, preventive strategies, and the prognosis of patients [7,88,92,93]. The exact cause of melanoma is not yet fully understood, but there are numerous factors that may initiate and promote its development: from ultraviolet (UV) radiation, genetic factors, geographical location, skin phototype, immunosuppression, viral infections [94], a high number of nevi, stress, biological/cytologic factors to gender. Despite the recent advances in the diagnosis and treatment of advanced melanoma, as stated in some studies, the best chance of survival is based on prevention/early detection [95,96,97].

## 5. Conclusions

Melanoma is an aggressive form of skin cancer that may occur spontaneously or through a linear progression from nevi under various key factors. Discovered in its early stages, it can be curable, with high survival rates, but with advanced/metastatic melanoma the patient’s prognosis worsens. There is hope that in the next years, there will be a better understanding of melanoma’s pathogenesis that will help discover it in its early stages and may aid in developing new effective treatments that could better the patient’s prognosis.

## Figures and Tables

**Table 2:** De novo melanoma (DNM) vs. nevus-associated melanoma (NAM), [6,40,78,85,86,87,88]. BI = Breslow index.

Melanoma Characteristics	
De Novo Melanoma	Nevus-Associated Melanoma
Prevalence—70–80%	Prevalence—20–30%
Spontaneous development	Develops on preexisting lesions (nevi)
Associated with—poor outcome, BI > 1 mm, nodular melanoma, located mostly on extremities, older age onset, tumor ulceration, low number of nevi.	Associated with—better prognosis, superficial spreading melanoma, younger age onset, fair skin, a high number of nevi, BI < 0.5 mm, BRAF^V600E^ mutation, tumors located on the trunk.

## Data Availability

The datasets used and/or analyzed during the present study are available from the corresponding author upon reasonable request.
